# Recent HIV infection among pregnant women in the 2017 antenatal sentinel cross–sectional survey, South Africa: Assay–based incidence measurement

**DOI:** 10.1371/journal.pone.0249953

**Published:** 2021-04-14

**Authors:** Selamawit Woldesenbet, Tendesayi Kufa-Chakezha, Carl Lombard, Samuel Manda, Mireille Cheyip, Kassahun Ayalew, Brian Chirombo, Peter Barron, Karidia Diallo, Bharat Parekh, Adrian Puren

**Affiliations:** 1 Center for HIV and STI, National Institute for Communicable Diseases, Johannesburg, South Africa; 2 School of Public Health, University of the Witwatersrand, Johannesburg, South Africa; 3 Biostatistics Unit, South African Medical Research Council, Cape Town, South Africa; 4 Biostatistics Unit, South African Medical Research Council, Pretoria, South Africa; 5 Department of Statistics, University of Pretoria, Pretoria, South Africa; 6 Strategic Information Unit, Center for Disease Control and Prevention, Pretoria, South Africa; 7 HIV and Hepatitis Program, World Health Organization, Pretoria, South Africa; 8 Laboratory Branch, Centers for Disease Control and Prevention South Africa, Pretoria, South Africa; 9 Division of Global HIV/AIDS, International Laboratory Branch, Centers for Disease Control and Prevention, Atlanta, Georgia, United States of America; 10 Division of Virology, School of Pathology University of the Witwatersrand, Johannesburg, South Africa; Albert Einstein College of Medicine, UNITED STATES

## Abstract

**Introduction:**

New HIV infection during pre-conception and pregnancy is a significant contributor of mother–to–child transmission of HIV in South Africa. This study estimated HIV incidence (defined as new infection within the last one year from the time of the survey which included both new infections occurred during pregnancy or just before pregnancy) among pregnant women and described the characteristics of recently infected pregnant women at national level.

**Methods:**

Between 1 October and 15 November 2017, we conducted a national cross–sectional survey among pregnant women aged 15–49 years old attending antenatal care at 1,595 public facilities. Blood specimens were collected from pregnant women and tested for HIV in a centralised laboratory. Plasma viral load and Limiting Antigen Avidity Enzyme Immunosorbent Assay (LAg) tests were further performed on HIV positive specimens to differentiate between recent and long–term infections. Recent infection was defined as infection that occurred within one year from the date of collection of blood specimen for the survey. Data on age, age of partner, and marital status were collected through interviews. Women whose specimens were classified as recent by LAg assay and with viral loads >1,000 copies/mL were considered as recently infected. The calculated proportion of HIV positive women with recent infection was adjusted for assay–specific parameters to estimate annual incidence. Survey multinomial logistic regression was used to examine factors associated with being recently infected using HIV negative women as a reference group. Age–disparate relationship was defined as having a partner 5 or more years older.

**Results:**

Of 10,049 HIV positive participants with LAg and viral load data, 1.4% (136) were identified as recently infected. The annual HIV incidence was 1.5% (95% confidence interval (CI): 1.2–1.7). In multivariable analyses, being single (adjusted odds ratio, aOR: 3.4, 95% CI: 1.8–6.2) or cohabiting (aOR: 3.8, 95% CI: 1.8–7.7), compared to being married as well as being in an age–disparate relationship among young women (aOR: 3.1, 95% CI: 2.0–4.7; reference group: young women (15–24years) whose partners were not 5 years or more older) were associated with higher odds of recent infection.

**Conclusions:**

Compared to previous studies among pregnant women, the incidence estimated in this study was substantially lower. However, the UNAIDS target to reduce incidence by 75% by 2020 (which is equivalent to reducing incidence to <1%) has not been met. The implementation of HIV prevention and treatment interventions should be intensified, targeting young women engaged in age–disparate relationship and unmarried women to fast track progress towards the UNAIDS target.

## Introduction

New maternal HIV infection among pregnant women contributes significantly to mother–to–child transmission of HIV (MTCT) [1–3]. In 2019, new maternal HIV infection during pregnancy was reported to be the second major cause of perinatal HIV transmission globally [4]. In 2011, new maternal HIV infection accounted for an estimated 26% of vertically transmitted early infant HIV infections in South Africa [5]. Pregnancy constitutes a period of elevated risk for HIV acquisition [6]. A study by Thomson et al. showed the per-act probability of HIV acquisition increased by 2.8 during pregnancy compared to during non-pregnancy period [6]. In a meta–analysis of studies conducted between 2014 and 2018, the pooled HIV incidence among pregnant women in sub–Saharan African and other countries was reported to be 2.1 per 100–person years [95% prediction interval(*prediction interval is a type of confidence interval used for prediction analysis)*: 0.7–6.5] [7].

The risk of MTCT in incident infections is increased from high maternal viral load during the early phase of HIV infection [8, 9]. Initiation of ART as early as possible in the course of new HIV infection can reduce maternal viral load and the risk of MTCT in newly acquired maternal HIV infection [10]. For this reason, the latest South African HIV testing guideline recommends that all pregnant women who test negative at initial antenatal care (ANC) testing should be offered repeat testing every 3 months throughout pregnancy and breastfeeding in order to ensure timely identification and treatment of new maternal HIV infections [11, 12].

There are also well–established strategies for prevention of maternal HIV infection during pregnancy. These include partner testing, condom use during pregnancy and breastfeeding, voluntary medical male circumcision (VMMC) for partners, couple testing prior to conception, provision of ART to partner with HIV, management of sexually transmitted infections (STI) and pre–exposure prophylaxis (PrEP) [13–15]. Despite these interventions, studies in sub–Saharan countries show a number of gaps in the coverage of HIV testing, ART and prevention services, including low knowledge of partner’s HIV status prior to conception, low coverage of HIV repeat testing during pregnancy, and high prevalence of risky behaviour (unprotected sex and multiple sexual partners), which can lead to new HIV acquisition [5, 16–18].

Monitoring the incidence of HIV among pregnant women and characterizing those infected during pre–conception and pregnancy can facilitate evaluation of interventions and evidence–based priority setting in implementing interventions targeting to reduce new HIV infection during pregnancy.

Most studies use a prospective cohort follow–up method (the reference method) to measure HIV incidence among pregnant women [19, 20]. Cohort methods are costly to implement at national level. Thus, current incidence estimates for pregnant women are based on meta–analyses of several small–scale cohort studies. Detection of biomarkers of recent infections from cross–sectional samples are an alternative and less expensive method to measure HIV incidence at national level. In this method, the incidence is estimated based on serological biomarkers in combination with clinical data e.g. viral load cut–off only or in combination with ART exposure. This combination of factors form the recent infection testing algorithm (RITA). While RITAs have been used and validated in the general population, the accuracy of these methods have not been assessed among pregnant women in settings with high prevention of MTCT (PMTCT) coverage [21–25].

This study used the biomarker method to measure HIV incidence among pregnant women nationally and described the characteristics of recently infected pregnant women using data from the 2017 Antenatal HIV Sentinel Survey in South Africa.

## Methods

The current study estimated the incidence of HIV among pregnant women enrolled in the 2017 South African Antenatal Sentinel HIV Survey using a RITA that included the Limiting Antigen Avidity Enzyme Immunosorbent Assay (LAg) and viral load measurement as markers in a serial algorithm for recent infection (RITA1– primary method) [21–24]. Sensitivity analysis was performed using a second method (RITA2) that included LAg, viral load and self–reported antiretroviral (ARV) exposure to estimate incidence and compare results between RITA1 and RITA2.

The South African Antenatal Sentinel HIV Survey is a cross–sectional survey, conducted every two years, to measure HIV prevalence among pregnant women attending ANC at public health care facilities in South Africa. The 2017 survey envisaged enrolment of 36,015 pregnant women, aged 15–49 years, from 1,595 public facilities selected from all 52 districts of South Africa. Public health facilities included in the survey were selected from each district using probability proportional to size sampling method. Consenting women were sampled consecutively from each selected facility until the required sample size was reached or until the end of the study, which ever one comes first. With the planned sample size for the 2017 survey, it was possible to estimate HIV incidence within a precision of 0.3% at national level. This precision was calculated based on previously recommended method [24], assuming expected incidence rate of 1.5%, mean duration of recent infection (MDRI) of 161 days (95% CI: 145–177 days), false recent rate (FRR) of 0 and time cut–off separating true recent from false recent infection (Big T) of 365 days.

The survey was conducted between 1 October and 15 November 2017. Data were collected through interviews and medical record review. Demographic information collected through interview included marital status, education, and partner’s (the father of the child) age. Data on age of the woman, HIV status (per point–of–care rapid testing) at first ANC visit or enrolment in the survey, and timing of ART initiation were extracted from medical records of enrolled women (if ART status was not documented on medical records, participants self–reported ART status was obtained). Blood specimens were tested for HIV (all specimens were tested regardless of prior knowledge of HIV–positive status); and if HIV positive, specimens were tested for recent infection, and viral load in the laboratory. Detailed description of site selection criteria, sampling of women, and the data collection procedures is presented elsewhere [26].

### Specimen testing for HIV

**Fig 1:**
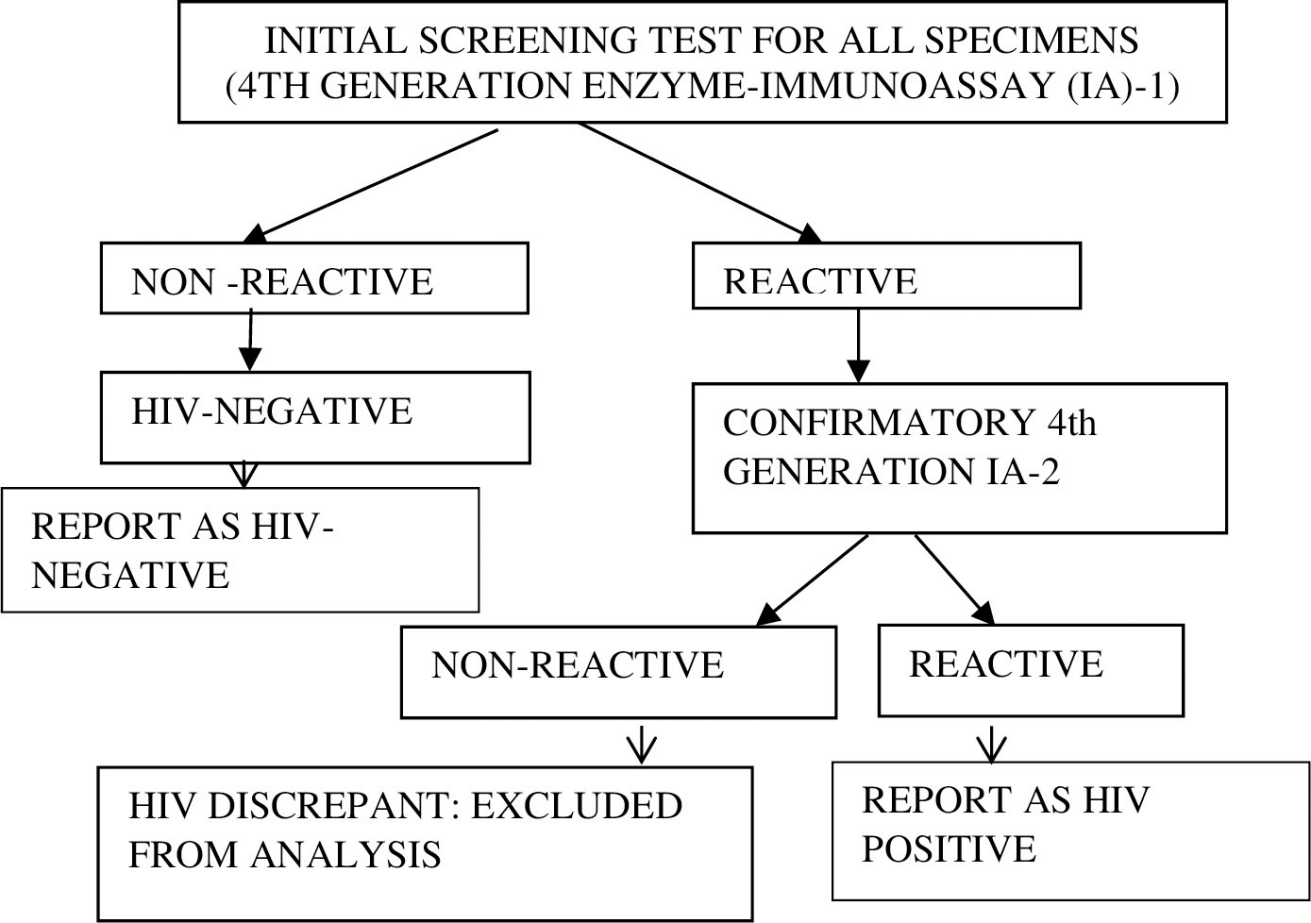
The laboratory HIV testing algorithm for the 2017 Antenatal HIV Sentinel Survey, South Africa.

At regional laboratories, specimens were tested for presence of HIV antibodies and antigens using a serial algorithm that consisted of two fourth generation enzyme–immunoassay (IA) platforms (Fig 1).

#### Identification of recent infections

Residual specimens from participants with a confirmed HIV–1 positive test result were tested for recency of infection using the LAg assay (SEDIA Biosciences, Portland, OR). A screening LAg test was first performed on all HIV positive samples. A specimen with a normalized optical density (ODn) of >2.0 on screening test was considered a long term infection. A long term infection was defined as infection duration of > 1 year. If the ODn was ≤2.0 the sample was re–tested in triplicate i.e. confirmatory testing was done, and if the median ODn of the triplicate confirmatory testing was <1.5, the sample was considered a recent infection. A recent infection was defined as infection that occurred within one year from the date of collection of blood specimen for the survey. If the sample ODn was ≥1.5 after confirmatory testing, it was considered a long–term infection. Western blotting was performed on specimens with an ODn ≤ 0.4 to confirm HIV status. Specimens with a negative or indeterminate Western blot result were reclassified as HIV negative and excluded.

**Fig 2:**
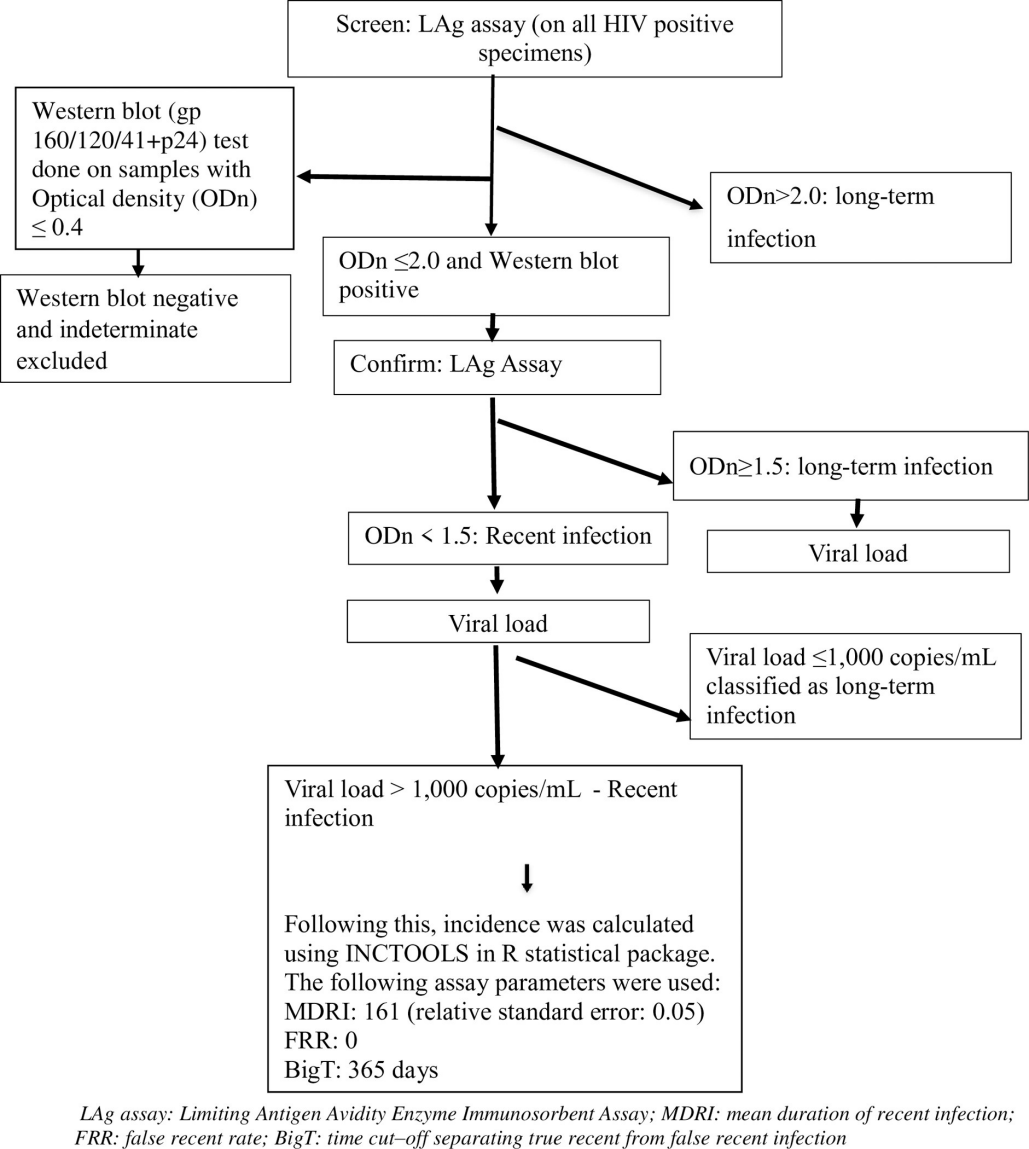
Recent infection testing algorithm (RITA1) in the 2017 Antenatal HIV Sentinel Survey, South Africa.

Two RITAs were used to differentiate recent infections from long–term infections. The first RITA consisted of the LAg result in combination with the HIV viral load result (Fig 2). In this RITA (RITA 1), specimens that were classified as LAg recent and with viral loads >1,000 copies/mL were defined as recently infected. In a separate sensitivity analysis, a viral load cutoff point of >400 copies/mL on RITA1 was used to differentiate recent infections from long-term infections.

In the second RITA (RITA2), a combination of LAg, self–reported ARV status, and viral load, was used. The findings of this sensitivity analysis (RITA2) is presented under S1 Section.

### Data analysis

The proportion of pregnant women who were HIV positive and the proportion of HIV positive women who met criteria for recent infection were estimated using STATA ® 14 (Stata Corporation. College Station, TX). Both these estimations and the logistic regression analysis described below took into account the survey design (clustering within health facilities, and stratification by district) and was benchmark weighted for the Statistics South Africa (Stats SA) 2017 mid–year population size of women of reproductive age (15–49 years) [26, 27].

HIV incidence was estimated as an annual instantaneous rate using the equation presented under S1 Equation [21, 28]. MDRI was set at 161 days, FRR was set at 0, and the relative standard errors for MDRI was set at 0.05[29, 30]. Sensitivity analysis was done changing the FRR value to 0.001. The big T was set at 365 days. This incidence estimation was implemented in R package (inctools) version 3.5.3 developed by the South African Centre for Epidemiological Modelling and Analysis, Stellenbosch University [21]. No previous study assessed the sensitivity and specificity of RITA among pregnant women. Given the lack of data, additionally, we conducted sensitivity analysis using varying levels of sensitivity (90–98%) and specificity (90–98%) to account for uncertainty in the specificity and sensitivity of the RITA method in the PMTCT setting (the findings of this sensitivity analysis is presented under S1 Table).

Descriptive statistics were used to compare the demographic and clinical characteristics of recently infected, long–term infected, and HIV negative participants. Age difference with partner was calculated by subtracting the age of the woman from her partner’s age. The age difference was dichotomized into an age difference of <5 years or ≥5 years. This age difference cutoff was informed by previous literature [31, 32].

Two survey domain based multivariable multinomial logistic regression models (using HIV uninfected and long–term infected individuals respectively as a reference group) were fitted to examine factors associated with recent infection. The second model fitted using long–term infected women as a reference group is presented under the S2 Section. Adjusted Odds ratio (aOR), and 95% CIs are reported from multivariable modelling. Variables significant in a univariable model at P value of ≤0.2 were included in a multivariable model. An interaction term between age difference with partner and age of the woman was included in the multivariable model, as the effect of age–disparate relationship is expected to be different for different age groups [33]. The significance of the interaction term was tested using a Wald test.

### Ethical considerations

Written informed consent was obtained from each participant prior to enrolment into the survey. Ethical approval was obtained from the University of the Witwatersrand Human Research Ethics Committee (Medical). The study protocol was also reviewed in accordance with the Centers for Disease Control and Prevention (CDC) human research protection procedures and was determined to be research, but CDC investigators did not interact with human subjects or have access to identifiable data or specimens for research purposes.

## Results

**Fig 3:**
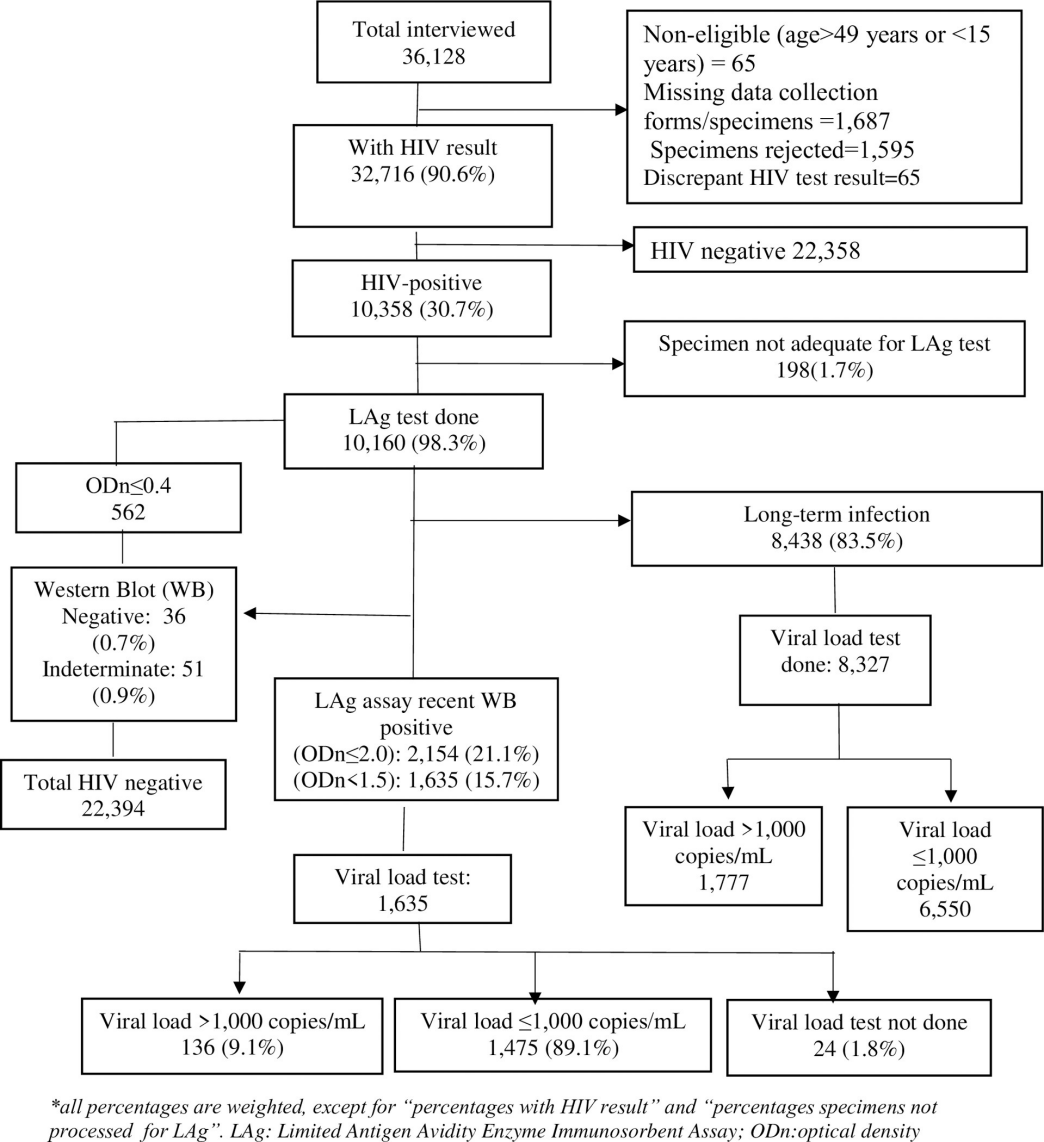
Recent infection algorithm, Antenatal HIV Sentinel Survey, 2017, South Africa.

A total of 36,128 participants were enrolled in the 2017 national antenatal sentinel HIV survey, of which 90.6% (32,716) had confirmed HIV results (Fig 3). Of those with confirmed HIV results, 30.7% (10,358) were HIV positive. LAg test was performed on 98.3% (10,160) of HIV positive specimens, and 16.5% (1,722) of LAg tested HIV positive specimens were confirmed as LAg recent (ODn<1.5). Western blot test performed on LAg tested specimens with an ODn ≤ 0.4 found 87 participants as HIV negative (36, 0.7%) or indeterminate (51, 0.9%). These specimens were re–categorized as HIV negative and indeterminate, respectively, and excluded from the analysis. Of the remaining 15.7% (1,635) participants with confirmed LAg recent results, 1.8% (24) participants’ specimens were not tested for viral load due to inadequate specimen and 89.1% (1,475) of participants had viral load ≤1,000 copies/mL, the later (i.e. participants with viral load ≤1,000 copies/mL) were considered long–term HIV infection. Therefore, of 10,049 HIV positive participants with LAg and viral load data, 1.4% (136) met criteria for recent infection using both the LAg assay and viral load algorithm *(Note*: *the denominator 10*,*049 was calculated as 10*,*060 LAg tested HIV positive participants minus 87 Western blot negative or indeterminate participants minus 24 participants for whom viral load test was not done*).

### HIV incidence

The estimated annual HIV incidence rate was 1.5% (95% CI: 1.2–1.7%) i.e. 1.5 cases per 100 person-years (S2 Table). Incidence was higher among first ANC visit attendees (1.8%, 95% CI: 1.4–2.2%) compared with follow–up–visit–attendees (1.1%, 95% CI: 0.9–1.4%) although this difference was not statistically significant. Incidence was not significantly different between younger (15–24 years) (1.5%, 95% CI: 1.2–1.9%) and older (25–49 years) pregnant women (1.4%, 95% CI: 1.1–1.6%). There was no statistically significant difference in incidence rate by gestational age (S2 Table). Changing the FRR value to 0.001 (instead of 0) reduced the recent HIV incidence estimate by 0.05% (to 1.45%). Changing the viral load cut-off in RITA 1 to >400 copies/mL increased the annual incidence estimate to 1.8% (95% CI: 1.5–2.0%). Additional sensitivity analysis showed the true incidence rate could range between 1.54% and 1.68% or 1.54% and 1.59% for sensitivity levels (of RITA 1) ranging 90–98% (with specificity level of 98%) and for specificity levels ranging 90–98% (with sensitivity level of 98%) respectively (S1 Table).

### Characteristics of participants who met criteria for recent infection

All recently infected participants were tested during ANC visit and the majority (68.7%, 87) learnt their HIV status during pregnancy, while 14.8% (25) were aware of their HIV positive status prior to pregnancy. Of the recently infected participants, 16.5% (20) were unaware of their HIV positive status, although they had tested during ANC (the ANC HIV test result of these participants was negative (14.5%) or discrepant (2.0%)) (Table 1).

**Table 1:** Characteristics of recently infected, long–term infected and HIV negative pregnant women in the 2017 Antenatal HIV Sentinel Survey, South Africa.

	Recently infected (n = 136)	HIV negative participants (n = 22,394*)	Long–term Infected (n = 9,913)	Significance of association (Chi2 test) with recent infection	
				Using HIV negative as reference group	Using long–term infected as reference group
	n (%)	n (%)	n (%)		
**Age (years)**					
15–24	71 (51.9)	10,467 (50.0)	2,367 (24.8)	0.6	<0.01
25–49	56 (48.1)	10,043 (50.0)	6,794 (75.2)		
**Marital status**					
Single	106 (80.1)	15,669 (70.6)	7,156 (73.0)	<0.01	0.02
Married	10 (7.7)	4,088 (19.7)	1,481 (15.7)		
Cohabiting	14 (12.2)	2,002 (9.5)	1,028 (10.8)		
Divorced/widowed	0	52 (0.2)	53 (0.5)		
**Partner age difference (in years)**					
Partner older by ≥5 years	56 (51.5)	6,002 (31.1)	3,209 (37.0)	<0.01	<0.01
Partner not older by ≥5 years	65 (48.5)	13,686 (68.9)	5,518 (63.0)		
**Education**					
None or primary	10 (9.4)	2,239 (10.7)	1,164 (12.1)	0.05	0.4
Secondary	109 (82.3)	16,399 (75.1)	7,530 (78.2)		
Tertiary	11 (8.3)	2,933 (14.2)	890 (9.7)		
**Visit type**					
First ANC visit	65 (53.1)	8,710 (41.6)	3,306 (35.5)	<0.01	<0.01
Follow–up ANC visit	66 (46.9)	13,347 (58.4)	6,422 (64.5)		
**Median (IQR) gestational age:**					
All	24 (17–30)	25 (17–32)	25 (17–32)	0.4	0.8
First ANC visit	19 (12–24)	16 (12–22)	16 (12–22)	0.2	0.2
Follow–up ANC visit	28 (24–34)	30 (24–34)	28 (22–34)	0.5	0.7
**Gravidity**					
Primi gravida	49 (33.2)	9,028 (40.2)	1,587 (15.8)	0.03	<0.01
Multi gravida	85 (66.8)	12,948 (59.8)	8,189 (84.2)		
**Province**					
Eastern Cape	22 (13.4)	2,681 (10.2)	1,302 (11.6)	<0.01	<0.01
Free State	10 (4.2)	1,839 (4.8)	872 (5.4)		
Gauteng	26 (33.1)	3,289 (26.1)	1,516 (28.3)		
KwaZulu–Natal	36 (20.1)	4,857 (16.9)	3,319 (27.1)		
Limpopo	11 (9.6)	2,033 (11.0)	511 (6.5)		
Mpumalanga	8 (5.0)	1,805 (7.1)	1,047 (9.7)		
North West	8 (1.6)	1,643 (2.1)	544 (1.6)		
Northern Cape	7 (6.9)	1,237 (7.6)	260 (3.8)		
Western Cape	8 (6.1)	2,986 (14.2)	538 (6.0)		
**ART status**					
Initiated ART prior to first ANC visit	19 (11.6)		5,421 (56.4)		<0.01
Initiated ART in prior ANC visit	23 (18.1)		2,087 (22.5)		
Initiated ART (date of ART initiation not known)	8 (5.9)		364 (3.8)		
Initiated on ART today	47 (39)		989 (11.5)		
Not on ART	34 (25.4)		531 (5.8)		
Median viral load (IQR)**(copies/mL)	18,016 (4,177–50,658)		208 (61–3,470)		<0.001
**Testing history (at ANC clinic)**					
Tested negative in previous ANC visit	8 (7.3)		190 (2.0)		<0.001
Tested negative on the day of the survey	9 (7.2)		93 (1.0)		
Positive before first ANC visit	25 (14.8)		6,079 (61.8)		
Tested positive on the day of the survey (at first ANC visit)	46 (38.2)		961 (10.9)		
Tested positive during ANC visit (*visit test done not known*)***	41 (30.5)		2,298 (24.2)		
Tested positive during ANC with discrepant result	3 (2.0)		9 (0.1)		

Missing data excluded; all percentages are weighted. IQR: inter–quartile range; ANC: antenatal care.

* These were tested negative either with enzyme–immuno assay (IA) (n = 22,358) or Western blot (n = 36).

** undetectable viral load excluded from the median /IQR estimates.

*** test may have been done in previous or current ANC visit.

Just under a third of recently infected participants had initiated ART in a prior ANC visit (18.1%) or before pregnancy (11.6%), while 39% initiated treatment on the day of the survey, and 25.4% were ART naïve (Table 1). The median viral load of recently infected participants was 18,016 copies/mL (IQR: 4,177–50,658 copies/mL) compared with median viral load of 208 copies/mL (61–3,470 copies/mL) for long–term infected participants, after excluding participants with undetectable (lower than detectable limit) viral load.

### Factors associated with recent HIV infection (reference group for comparison: HIV negative)

In a multivariable analysis (adjusting for gravidity and education), marital status, and age–disparate relationship among young women (15–24 years) were associated with recent infection. Among women aged 15–24 years, those with partners’ ≥ 5 years older were 3.1 times (aOR: 3.1, 95% CI: 2.0–4.7) more likely to be recently infected compared with women in the same age group whose partners were not 5 years or more older (Table 2). We found interaction between age and age–disparate relationship (P value for interaction term: 0.045). Age–disparate relationship was not influential on recency among older women. After adjusting for age–disparate relationship, age was not influential on recency of HIV infection. Single (aOR: 3.4, 95% CI: 1.8–6.2) and cohabiting (aOR: 3.8, 95% CI: 1.8–7.7) women had higher odds of recent infection compared with married women. The odds of recent infection were higher among first ANC visit attendees compared with follow–up visit attendees (aOR: 1.6, 95% CI: 1.2–2.2).

**Table 2:** Factors associated with recent HIV infection (reference group for comparison: HIV negative) in the 2017 Antenatal HIV Sentinel Survey, South Africa.

	N (%)	Unadjusted OR (95% CI)	Adjusted OR (95% CI)
N = 32,716			
**Marital status**			
Single	23,255 (71.7)	2.9 (1.7–5.1)	3.4 (1.8–6.2)
Cohabiting	3,060 (9.8)	3.3 (1.7–6.5)	3.8 (1.8–7.7)
Married	5,633 (18.5)	1.0	1.0
**Visit type**			
First ANC visit attendees	12,322 (40.3)	1.6 (1.2–2.1)	1.6 (1.2–2.2)
Follow–up ANC visit attendees	19,898 (59.7)	1.0	1.0
**Age gap with partner among 15–24 years**			
Partner older by ≥5 years	4,363 (35.6)	3.1 (2.1–4.7)	3.1 (2.0–4.7)
Partner not older by ≥5 years	8,090 (64.4)	1.0	1.0
**Age gap with partner among 25–49 years**			
Partner older by ≥5 years	4,983 (31)	1.7 (1.1–2.8)	1.6 (0.9–2.6)
Partner not older by ≥5 years	11,320 (69)	1.0	1.0
**Age of woman (among women with partner** ≥**5 years older)**			
15–24 years	4,363 (45.9)	1.4 (0.9–2.2)	1.4 (0.8–2.3)
25–49 years	4,983 (54.2)	1.0	1.0
**Age of woman (among women with partner not older by** ≥**5 years)**			
15–24 years	8,090 (40.8)	0.8 (0.5–1.2)	0.7 (0.5–1.1)
25–49 years	11,320 (59.2)	1.0	1.0

Missing values excluded from logistic regression. N = 27,226 observations (83.2% of data) included in multivariable analysis. P value for interaction between age and age–disparate relationship = 0.045. The section of the multinomial model comparing HIV negative participants with long–term infected was not presented in this table as this was not the primary interest of this study. OR: odds ratio; ANC: Antenatal care. This model is adjusted for gravidity and education.

## Discussion

In this national study of HIV incidence among pregnant women, HIV incidence was estimated at 1.5% (i.e. 1.5 cases per 100 person-years) in 2017. The risk of new HIV infection was significantly higher among young women (15–24 years) in age–disparate relationships, single and cohabiting women, and women attending their first ANC visit (compared with follow–up visit attendees) at enrolment.

The incidence reported in this study was lower compared to most previous incidence estimates reported for pregnant women in South Africa [19, 20, 34–37] and elsewhere in neighbouring countries with a similar HIV burden [38]. The incidence reported in other studies in Africa average at 4.1 per 100 person–years (95% predictive interval: 1.1–12.2) in the years prior to 2010 and 2.1 person–years (95% predictive interval: 0.7–6.5) since 2014 [7]. The incidence reported in the current study was comparable with some of the lowest HIV incidence rates reported in intervention–based studies in South Africa, such as the study by Fatti et al. which reported incidence rate of 1.5% during pregnancy after a 4–year implementation of selected combination prevention interventions [39]. While the lower incidence observed among pregnant women in this study could be partially explained by the progress in the scale–up of combination prevention interventions, the study may also have underestimated the incidence rate in this population due to methodological limitations.

The measured incidence could have been lower than actual incidence due to misclassification bias associated with the use of viral load measurements in the algorithm for recent infection which categorizes all women with viral load ≤1,000 copies/mL as long–term infected. Viral load was included in the criteria for the RITA used in this study to reduce ‘false recent’ misclassification [40]. Prior to the introduction of test–and–treat, this algorithm worked well for estimating incidence as the guideline at the time recommended delayed ART initiation. While the scale up of test and treat has been a major breakthrough and an important intervention globally, as the number of individuals who initiate ART in the early stage of their infection increases with the scale up of test and treat, clinical criteria such as viral load and ART can no longer accurately differentiate recent infections from long–term infections [41, 42]. The PMTCT programme, in particular, has high ART coverage [43, 44]. Our finding of lower incidence rate among follow–up visit attendees (compared to first ANC visit attendees) substantiates our argument that the high uptake of ART at first ANC visit facilitates viral suppression in follow–up visits among recently infected women, which results in under estimation of incidence among follow–up visit attendees in the RITA method. As the coverage of initiation of ART increases with test–and–treat, assays which identify recent infections independently of viral load levels will be important as relying on viral load for identifying recent infections in high ART coverage settings will result in underestimation of incidence. In addition, to improve estimates in the current method, RITA should include clinical history such as new diagnosis, so that individuals with new diagnosis, if LAg–recent, will not be reclassified as long–term infected irrespective of viral load suppression and presence of ARV.

According to Thembisa modelling, incidence rate was 2.6% in 2010 among young women (15–24 years) in South Africa [45]. Compared to the Thembisa estimate, the incidence reported for young women (15–24 years) in our study, even if underestimated, represents at best a 42.1% decline in incidence between 2010 and 2017. While this decline reflects substantial progress, with the current pace, it will not be possible to reach the UNAIDS target of 75% decline in incidence (or <1% incidence rate) by 2020 [45].

Despite the use of different methods for estimating incidence, the population group identified to have higher risk in this study were consistent with those found in other cohort studies from South Africa [19, 20, 34, 36, 46] and other sub–Saharan African countries [47, 48]. These studies reported age–disparate relationship and being single or being in a cohabiting (non-marital) relationship to be among the main risk factors of new HIV infection among pregnant women as well as women in the general population. These findings underscore the importance of fast–tracking combination prevention interventions which provide alternatives for prevention of HIV infection during pregnancy and breastfeeding. In South Africa, although there are effective prevention interventions, the coverage of most prevention interventions, including couples testing, condom use during pregnancy and coverage of VMMC, is low [16–18]. The reasons why coverage of these interventions may be low include the interventions requiring male partner involvement for successful implementation, and low marriage or stable relationship rates in our population [49, 50]. To address this gap, in 2017, World Health Organization (WHO) recommended PrEP to be offered to pregnant women at substantial risk of HIV acquisition as an additional, female–controlled, prevention option [13]. South Africa has also recently approved PrEP for pregnant women [15]. The provision of PrEP for high–risk women at ANC clinics could substantially reduce new HIV acquisition during pregnancy and postpartum as well as vertical transmission of HIV [51]. This study has identified risk factors that should be considered in prioritizing women for PrEP. These risk factors along with other risk factors identified in earlier studies [38, 52] should be used to identify which pregnant women should be offered PrEP.

The South African national household survey conducted in the same year as this survey used RITA2 (LAg, viral load and ART) to estimate incidence among reproductive age women as opposed to RITA 1 (LAg and viral load) used in this study [53]. Despite the difference in the algorithm and target population, the incidence rate reported for young pregnant women (15–24 years) in our survey (1.5%) was identical to the incidence reported for young (15–24 years) women in the general population in the South African household survey. In our survey, using RITA 2 strategy (see S1 Section) resulted in substantial underestimation of incidence as all known HIV positive women were initiated on ART at first ANC visit in the PMTCT programme. Using the >400 copies/mL (instead of >1000 copies/mL) cut-off for viral load in the RITA 1 method increased the incidence estimate to 1.8. Although reducing the viral load cut-off to 400 copies/mL may improve sensitivity, this method could also reduce specificity. Future studies should find markers/tests that maximize both sensitivity and specificity.

This study showed gaps in early identification and initiation of ART among recently infected pregnant women. At a median gestational age of 24 weeks, a quarter of recently infected women were not initiated on ART. Most of these women were unaware of their HIV positive status despite testing during ANC visits. Repeat testing of HIV negative women in subsequent ANC visits should be strengthened per the guideline to identify new seroconversions early.

The study has some limitations. The study collected limited data on demographic, biological and behavioural factors which may explain differences in characteristics between long–term infected, recently infected, and HIV negative participants. We observed similar recent incidence rates in young (15–24 years) and older (25–49 years) women. This could be due to the under representation of older women (>35 years) in the survey as around 90% of participants in the antenatal survey were women younger than 35 years.

The overall percentage of lost data collection forms and specimens was small (4.7%). These lost data collection forms and specimens are less likely to introduce bias as the missingness of forms/specimens was not associated with the characteristics of participants or the outcome variable, rather it reflected gaps in logistics and training, and it affected all provinces. The incidence among pregnant women is likely to be higher than the incidence among 15–24 years old women in the general population. Therefore, the estimated decline in incidence between 2010 and 2017 (calculated using the 2010 Thembisa data and the current study) may underestimate the true decline in incidence among pregnant women.

## Conclusion

Among this population of pregnant women, this was the first attempt to estimate incidence at a national level. Compared to previous studies among pregnant women, the incidence estimated in this study was substantially lower. However, a steeper decline in HIV incidence is needed to achieve the UNAIDS target to reduce incidence by 75% by 2020. The implementation of HIV prevention and treatment interventions need to be intensified, targeting young women engaged in age–disparate relationship and unmarried women to fast track progress towards the UNAIDS target. Scaling up HIV testing and prevention interventions, including PrEP for high–risk populations such as adolescent girls and young women, couple HIV testing prior to conception, repeat testing during pregnancy, VMMC and ART coverage among men could help to fast track progress towards the UNAIDS target. These interventions are beneficial during both pregnancy and postpartum periods as the risk of new maternal infection continues to the postpartum period. Incidence may have been underestimated in this study as high ART coverage in the ANC setting could result in misclassification of virally suppressed recently infected women as long–term infection. As pregnant women are high–risk population group, it will be important to continue close monitoring of this population. To improve the accuracy of estimates from cross–sectional surveys, reliable assays which identify recent infections independently of viral load levels are needed.

## Supporting information

S1 Table HIV incidence for varying sensitivity and specificity levels of the RITA method in the PMTCT setting. (DOCX)

S2 Table Annual recent HIV incidence among pregnant women in South Africa in the 2017 Antenatal HIV Sentinel Survey using the LAg and viral load algorithm. (DOCX)

S1 Section Sensitivity analysis using RITA 2. (DOCX)

S2 Section Factors associated with recent HIV infection (reference group for comparison: Long–term infected). (DOCX)

S1 Equation Incidence estimation. (DOCX)
