# Supplementary material for: Recent HIV infection among pregnant women in the 2017 antenatal sentinel cross–sectional survey, South Africa: Assay–based incidence measurement
Source: PLoS One. 2021 Apr 14;16(4):e0249953. doi: 10.1371/journal.pone.0249953 (PMC8046194; doi:10.1371/journal.pone.0249953)
Supplement: S1 Table — (DOCX) [file pone.0249953.s001.docx]

**S1 Table.** HIV incidence for varying sensitivity and specificity levels of the RITA method in the PMTCT setting.

| Incidence  in current study (%) | Assumptions regarding misclassification bias | | Incidence after adjusting for misclassification bias* (%) |
| --- | --- | --- | --- |
|  | Sensitivity | Specificity |  |
| 1.5 | 0.98 | 0.98 | 1.54 |
| 1.5 | 0.97 | 0.98 | 1.56 |
| 1.5 | 0.96 | 0.98 | 1.57 |
| 1.5 | 0.95 | 0.98 | 1.59 |
| 1.5 | 0.90 | 0.98 | 1.68 |
| 1.5 | 0.98 | 0.98 | 1.54 |
| 1.5 | 0.98 | 0.97 | 1.55 |
| 1.5 | 0.98 | 0.96 | 1.55 |
| 1.5 | 0.98 | 0.95 | 1.56 |
| 1.5 | 0.98 | 0.90 | 1.59 |

* Incidence was calculated using the formula: Incidence_true = (Incidence_detected + specificity – 1)/(sensitivity + specificity – 1)
