# Supplementary material for: Recent HIV infection among pregnant women in the 2017 antenatal sentinel cross–sectional survey, South Africa: Assay–based incidence measurement
Source: PLoS One. 2021 Apr 14;16(4):e0249953. doi: 10.1371/journal.pone.0249953 (PMC8046194; doi:10.1371/journal.pone.0249953)
Supplement: S2 Table — (DOCX) [file pone.0249953.s002.docx]

**S2 Table.** Annual recent HIV incidence among pregnant women in South Africa in the 2017 Antenatal HIV Sentinel Survey using the LAg and viral load algorithm.

|  | **Annual recent HIV incidence**  % (95% CI) |
| --- | --- |
| **Overall** | 1.5 (1.2–1.7) |
| **Visit type**  First ANC visit attendees (n=8,775)  Follow–up visit attendees (n=14,413) | 1.8 (1.4 –2.2)  1.1 (0.9–1.4) |
| **Age**  15–24 years (n=10,538)  25–49 years (n=10,099) | 1.5 (1.2–1.9)  1.4 (1.1–1.6) |
| **Gestational age**  1^st^ trimester (n=3,045)  2^nd^ trimester (n=9,752)  3^rd^ trimester (n=8,662) | 1.4 (0.9–2.0)  1.7 (1.3–2.1)  1.2 (0.9–1.5) |

*n is the denominator for incidence which is the number of susceptible (negative) woman plus recently infected women ; LAg: Limiting Antigen Avidity Enzyme Immunosorbent Assay; CI: confidence interval; ANC: antenatal care*
