# Supplementary material for: Recent HIV infection among pregnant women in the 2017 antenatal sentinel cross–sectional survey, South Africa: Assay–based incidence measurement
Source: PLoS One. 2021 Apr 14;16(4):e0249953. doi: 10.1371/journal.pone.0249953 (PMC8046194; doi:10.1371/journal.pone.0249953)
Supplement: S1 Section — (DOCX) [file pone.0249953.s003.docx]

**S1 Section. Sensitivity analysis using RITA 2.**

In a sensitivity analysis performed, participants who were recent by LAg and had unsuppressed viral load (>1000 copies/mL) and no exposure to ART were classified as recently infected; participants were classified as long–term infected if they were identified as long–term infected by LAg test or if they had suppressed viral load (≤1000 copies/mL) or if they were virally unsuppressed but self–reported exposure to ART[[49](#_ENREF_49)].

In this analysis, incidence was 0.9% (95% CI: 0.7%–1.1%) in the overall sample. Incidence rate was the same between younger (15–24 years) (0.9%, 95% CI: 0.7%–1.2%) and older (35–49 years) (0.9%, 95% CI: 0.6–1.1%) women, but incidence stayed lower among follow–up ANC visit attendees (0.4%, 95%CI: 0.2%–0.5%) compared with first ANC visit attendees (1.6%, 95% CI: 1.2%–2.0%).

Estimating incidence with the above (RITA 2) strategy reduced incidence significantly. RITA 2 considers all women exposed to ART as long–term infected. This results in substantial underestimation of incidence among PMTCT women as all known HIV positive women are initiated on ART at first ANC visit in the PMTCT programme.
