# Supplementary material for: Recent HIV infection among pregnant women in the 2017 antenatal sentinel cross–sectional survey, South Africa: Assay–based incidence measurement
Source: PLoS One. 2021 Apr 14;16(4):e0249953. doi: 10.1371/journal.pone.0249953 (PMC8046194; doi:10.1371/journal.pone.0249953)
Supplement: S2 Section — (DOCX) [file pone.0249953.s004.docx]

**S2 Section. Factors associated with recent HIV infection (reference group for comparison: long–term infected).**

In a multivariable analysis that adjusted for education, recently infected women compared to long–term infected women, were more likely to be in age–desperate relationship (adjusted odds ratio – aOR: 1.6, 95% CI: 1.2–2.2) and co–habiting with their partner (aOR: 2.5, 95% CI: 1.2–5.2). Participants with recent infection were also more likely to be primigravida (AOR: 1.9, 95% CI: 1.4–2.7), young (15–24years) (aOR: 2.3, 95% CI: 1.7–3.3), and first ANC visit attendees (aOR: 2.1, 95% CI: 1.6–2.9) compared to long–term infected participants (Table 3).

Table 1: Factors associated with recent HIV infection (reference group for comparison: long–term infected) in the 2017 Antenatal HIV Sentinel Survey, South Africa

|  | **Unadjusted OR (95% CI)** | **Adjusted OR (95% CI)** |
| --- | --- | --- |
|  | **N=32,716** | |
| **Gravidity** |  |  |
| Primigravida | 2.6 (2.0–3.5) | 1.9 (1.4–2.7) |
| Multigravida | 1.0 | 1.0 |
| **Marital status** |  |  |
| Single | 2.2 (1.3–3.9) | 1.6 (0.9–3.0) |
| Cohabiting | 2.3 (1.2–4.6) | 2.5 (1.2–5.2) |
| Married | 1.0 | 1.0 |
| **Visit type** |  |  |
| First ANC visit attendees | 2.1 (1.5–2.7) | 2.1 (1.6–2.9) |
| Follow–up ANC visit attendees | 1.0 | 1.0 |
| **Age gap with partner** | | |
| >5 years | 1.8 (1.3–2.4) | 1.6 (1.2–2.2) |
| ≤ 5 years | 1.0 | 1.0 |
| **Age of the woman** | | |
| 15–24 years | 3.3 (2.5–4.3) | 2.3 (1.7–3.3) |
| 25–49years | 1.0 | 1.0 |

*Missing values excluded from logistic regression. N=27,226 observations (83.2% of data) included in multivariable analysis. The interaction between age and age–disparate relationship was not significant (P value 0.352), therefore interaction was removed from this model. The section of the multinomial model comparing HIV negative participants with long–term infected was not presented in this table as this was not the primary interest of this study. OR: odds ratio; ANC: Antenatal care ; CI confidence interval*
