# Supplementary material for: Recent HIV infection among pregnant women in the 2017 antenatal sentinel cross–sectional survey, South Africa: Assay–based incidence measurement
Source: PLoS One. 2021 Apr 14;16(4):e0249953. doi: 10.1371/journal.pone.0249953 (PMC8046194; doi:10.1371/journal.pone.0249953)
Supplement: S1 Equation — (DOCX) [file pone.0249953.s005.docx]

**S1 Equation. Incidence estimation.**

**Where**

**= Incidence  = number of recent infection = number of HIV positive (both recent and non–recent) ; ^= number of HIV negative^; ^= MDRI^, = the FRR and = time cut–off separating true recent from false recent infection**

* Equation taken from *Moyo S, Gaseitsiwe S, Mohammed T, Pretorius Holme M, Wang R, Kotokwe KP, et al. Cross–sectional estimates revealed high HIV incidence in Botswana rural communities in the era of successful ART scale–up in 2013–2015. PLoS One. 2018;13(10):e0204840.*
